# Supplementary material for: The phased pan-genome of tetraploid European potato
Source: Nature. 2025 Apr 16;642(8067):389–97. doi: 10.1038/s41586-025-08843-0 (PMC12158759; doi:10.1038/s41586-025-08843-0)
Supplement: Supplementary file 5 — Supplementary Figs. 3–14, 20, 21, 23–27 and 34–44. [file 41586_2025_8843_MOESM5_ESM.zip › suppl_figure_26.pdf]

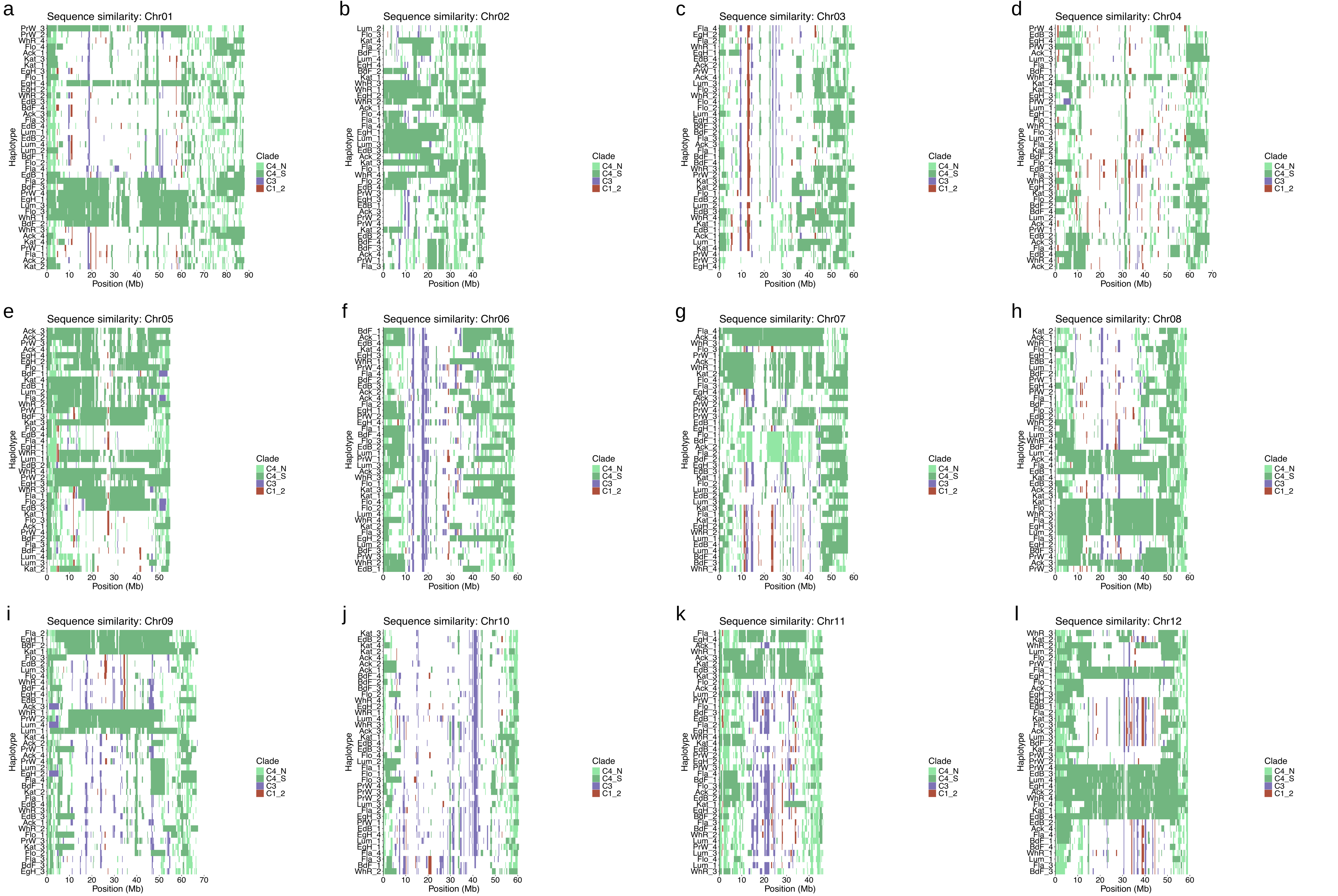

a
Sequence similarity: Chr01
b
Sequence similarity: Chr02
c
Sequence similarity: Chr03
d
Sequence similarity: Chr04
e
Sequence similarity: Chr05
f
Sequence similarity: Chr06
g
Sequence similarity: Chr07
h
Sequence similarity: Chr08
i
Sequence similarity: Chr09
j
Sequence similarity: Chr10
k
Sequence similarity: Chr11
l
Sequence similarity: Chr12
Haplotype
Position (Mb)
Clade
C4_N
C4_S
C3
C1_2
